# Molecular Dynamic Simulation of Primary Damage with Electronic Stopping in Indium Phosphide

**DOI:** 10.3390/nano14211738

**Published:** 2024-10-30

**Authors:** Yurong Bai, Wenlong Liao, Zhongcun Chen, Wei Li, Wenbo Liu, Huan He, Chaohui He

**Affiliations:** 1 School of Nuclear Science and Technology, Xi’an Jiaotong University, Xi’an 710049, China; baiyur@stu.xjtu.edu.cn (Y.B.); lwl1551528661@stu.xjtu.edu.cn (W.L.); chenzhongcun@stu.xjtu.edu.cn (Z.C.); nuclear@stu.xjtu.edu.cn (W.L.); hechaohui@xjtu.edu.cn (C.H.); 2 Science and Technology on Analog Integrated Circuit Laboratory, Chongqing 400060, China; 3 China Nuclear Power Technology Research Institute Co., Ltd., Shenzhen 518000, China

**Keywords:** indium phosphide, primary damage, electronic stopping, molecular dynamics simulation

## Abstract

Indium phosphide (InP) is an excellent material used in space electronic devices due to its direct band gap, high electron mobility, and high radiation resistance. Displacement damage in InP, such as vacancies, interstitials, and clusters, induced by cosmic particles can lead to the serious degradation of InP devices. In this work, the analytical bond order potential of InP is modified with the short-range repulsive potential, and the hybrid potential is verified for its reliability to simulate the atomic cascade collisions. By using molecular dynamics simulations with the modified potential, the primary damage defects evolution of InP caused by 1–10 keV primary knock-on atoms (PKAs) are studied. The effects of electronic energy loss are also considered in our research. The results show that the addition of electronic stopping loss reduces the number of point defects and weakens the damage regions. The reduction rates of point defects caused by electronic energy loss at the stable state are 32.2% and 27.4% for 10 keV In-PKA and P-PKA, respectively. In addition, the effects of electronic energy loss can lead to an extreme decline in the number of medium clusters, cause large clusters to vanish, and make the small clusters dominant damage products in InP. These findings are helpful to explain the radiation-induced damage mechanism of InP and expand the application of InP devices.

## 1. Introduction

As a III–V compound semiconductor, InP is widely applied in electronic devices and radiation environments for its high electron mobility, direct band gap, and high-temperature resistance [1]. However, many experiments have demonstrated that the defects induced by radiation particles (e.g., electron, neutron, proton, or heavy ion) lead to the degradation of InP devices [2,3,4,5,6,7,8], which is called the displacement damage (DD) effect. Therefore, understanding the physics of defect production, stabilization, and clustering is necessary for predicting the degree of displacement damage and enhancing the useable lifetime of InP devices in high-radiation environments. 

Yamaguchi et al. [1] found that the introduction of the major hole trap center H4 (E_V_ + 0.37 eV) caused InP solar cell property degradation with irradiation, and minority-carrier injection into InP annihilates the H4 center. They predicted the H4 center in InP introduced by electron irradiation at room temperature to be associated with a point defect such as phosphorus vacancy (V_p_). Chen et al. [9] used the density functional theory (DFT) to verify the ε(0/−) charge-transfer level of V_p_ at E_V_ + 0.33 eV, which is consistent with the experimental charge-state assignment of H4. Meanwhile they explained that the injection-enhanced annealing of H4 is caused by the recombination of V_p_ and a mobile phosphorus interstitial (P_i_), which is shown to rapidly diffuse with a migration barrier of 0.04 eV under minority-carrier injection. Liu et al. [10] discovered that the two electron traps E5 (E_C_ − 0.66 eV) and E6 (E_C_ − 0.68 eV) generated by the incident of 2 MeV electrons on InP [11] are ascribed to the transition energy levels ε(−1/−2) and ε(−2/−3) of In vacancies (V_In_) by using DFT.

DFT simulations of InP generally concentrate on calculating the basic properties of defects, such as the defect formation energy, transition energy level, and migration energy. However, the size of systems (about 200 atoms) is limited due to computational resources, which cannot describe the entire process of displacement damage. The molecular dynamics (MD) method is often applied to explore the atomistic displacement cascades of the large system with a million atoms in III–V compound semiconductors, such as GaAs [12,13], GaN [14,15], SiC [16,17], and InAs [18]. However, research on displacement cascades in InP is hindered by the scarcity of accurate interatomic potential. Although Branicio et al. [19] fitted parameters based on an interatomic potential model [20] to predict the thermal and mechanical properties of InP, this potential has limited applicability in the case of native points defects simulations. Recently, Chrobak et al. [21] developed new parameters of InP based on the analytical bond-order potential (ABOP), while only the formation energy of InP native point defects is studied and the short-range repulsive force is not considered in this potential, which is proven to be significant in simulating displacement cascade [22]. 

Therefore, we optimize the ABOP potential of InP by connecting it with the short-range repulsive potential to accurately describe atomic cascade collisions. Moreover, the feasibility of the modified potential is confirmed by the basic physical properties and the quasi-static simulations. By using MD with the modified potential, the evolution and spatial distribution of point defects in InP induced by 1~10 keV In-PKA and P-PKA at room temperature are discussed first. The electronic stopping (ES) is also considered for a complete model of collision cascades [23,24,25,26,27,28,29]. Moreover, the mechanism of both vacancy and interstitial clusters evolution and the number of clusters at stable state are studied to explore the influence of ES on the accumulation of point defects. Our study demonstrates the significance of ES effects in radiation-damage cascades and helps us understand how InP responds to a severe radiation environment.

## 2. Materials and Methods

### 2.1. Molecular Dynamics Modeling

LAMMPS code (Large-scale Atomic/Molecular Massively Parallel Simulator) [30] of version 28Mar2023 is utilized to investigate the displacement damage simulations of InP with the zinc blende structure. To prevent the PKA from leaving the system, the cell size is carefully chosen for 1~10 keV In-PKA and P-PKA, as shown in Table 1.

To achieve the equilibration of the system, the conjugate gradient method is used on the simulation box to evolve to the state with the lowest energy. Then, the total system is further equilibrated with canonical assemblage (NVT) at 300 K for 1 ps. Subsequently, the inner layer of the box is maintained under NVE, while the ensemble of the outer layer (one-tenth thick of the system) is maintained as NVT to ensure the simulation domain at 300 K. This period lasts 1 ps. Afterward, one atom in the middle of the cell is chosen as the PKA to define its energy, and fifteen PKA events are performed at each energy with random direction to achieve a good statistic. A variable timestep is employed to allow for a small value in the early binary collision and for a large value in the remaining primary damage (from 0.001 ps to 1 ps). The total time of collision cascades is 17.5 ps.

Point defects (Frenkel pairs, antisites) are recognized in the simulations using an improved Wigner–Seitz (WS) cell analysis of the atom positions [12]. A Frenkel pair (FP) is formed when an atom from a regular lattice site moves to an interstitial position within the crystal, leaving behind an unoccupied lattice site, or vacancy. An antisite defect occurs when an atom occupies a lattice site that is normally occupied by a different type of atom in the crystal structure, such as In_p_ or P_In_. For the definition of clusters, it has been reported that there is no attractive force to form clusters between two defects when their distance is out of the second-nearest-neighbor distance [12]. Therefore, the module “Cluster Analysis” of OVITO [31] is utilized to verify the clusters when the distance between two point defects is within 4.12 Å. Clusters are divided into three categories based on the number of point defects they contain: (1) small clusters (1–10 point defects), (2) medium clusters (11–30 point defects), and (3) large clusters (more than 31 point defects). 

To better describe the physics of cascades, the electronic stopping is implemented by applying a friction force to each atom as follows:

(1) $$\vec{F_{i}}=\vec{F_{i}^{0}}-\frac{\vec{v_{i}}}{\left|\left|\vec{v_{i}}\right|\right|}\;S_{e}$$

where $\vec{F_{i}}$ is the resulting total force on the atom, $\vec{F_{i}^{0}}$ is the original force applied to the atom, $\vec{v_{i}}$ is its velocity, and $S_{e}$ is the electronic stopping power of PKA. In InP, $S_{e}\;$ is predicted by “Stopping and Range of Ions in Matter” SRIM 2013 code [32].

The reduction rate of defect numbers is calculated to measure the impact of ES, and the formula is as follows:

(2) $$D=\frac{N_{without\;ES}-N_{with\;ES}}{N_{without\;ES}}$$

where $N_{without\;ES}$ and $N_{with\;ES}$ are the number of defects calculated by MD without and with ES, respectively.

### 2.2. Interatomic Potential

The ABOP potential has been applied extensively to simulate defect properties and defect creation in numerous semiconductors [18,33,34,35]. In this work, the interactions between In and In, In and P, and P and P can be described by the ABOP potential parametrized by Chrobak et al. [21]. The ABOP potential of InP has well described the reversibility of the pressure-induced B3 ↔ B1 phase transition as well as the formation of native point defects in the B3 phase, enabling atomistic computer simulations to cover a wide range of materials problems related to InP. However, the ABOP potential alone still cannot well construct the short-range interactions which are important for simulating atomic displacement and defect creation at high-energy PKA. To overcome this shortage, the Ziegler–Biersack–Littmark (ZBL) [36] screen potential is connected with the ABOP potential in LAMMPS, which is more suitable for describing the physical process of small atomic collision in primary displacement damage. The hybrid potential is called the ABOP + ZBL potential of InP, which is achieved by using the Fermi-type function:

(3) $$F\left(r\right)=\frac{1}{1+e^{{-b}_{f}(r-r_{f})}}$$

where *F*(*r*) quickly goes to one as r increases. The total potential is given by connecting ABOP to ZBL potential:

(4) $$V\left(r\right)=V_{ZBL}\left[1-F\left(r\right)\right]+V_{ABOP}F(r)$$

which yields a repulsive potential dominated by the ZBL potential for a very short distance and quickly approaches the ABOP potential as r increases. The parameters of $b_{f}$ and $r_{f}$ are adjusted to ensure that the potential and its first derivative are smoothly transitioned. The values of $b_{f}$ are determined to be 14.5, 14.5, and 7.8 Å^−1^, and those of $r_{f}$ are 0.99, 0.99, and 0.67 Å for In–In, In–P, and P–P interactions, respectively. To verify its accuracy by comparing with the DFT results, the hybrid potential is used to calculate the basic physical properties and point defect formation energy of InP in Section 3.1 while the quasi-static calculations of InP are simulated in Section 3.2.

The formation energy of a defect is defined as the energy difference between the system with the defect and a perfect (minimum-energy) system with the same composition; its calculation requires the use of the chemical potentials μ to adjust the stoichiometry of the perfect system. The formation energies $E_{f}\;$ of $V_{p}$ and $V_{In}$ are calculated as follows:

(5) $$E_{f}\left(V_{p}\right)=E\left(V_{p},{In}_{N}P_{N-1}\right)-E\left({In}_{N}P_{N}\right)+\mu_{p}$$

(6) $$E_{f}\left(V_{In}\right)=E\left(V_{In},{In}_{N-1}P_{N}\right)-E\left({In}_{N}P_{N}\right)+\mu_{In}$$

where N is the number of atoms with each species in the supercell. $E\left({In}_{N}P_{N}\right)$ is the calculated total energy of the perfect supercell. $E\left(V_{p},{In}_{N}P_{N-1}\right)$ and $E\left(V_{In},{In}_{N-1}P_{N}\right)$ are the (relaxed) energiesof the supercell containing single $V_{p}$ and $V_{In}$, respectively. Similarly, $E_{f}$ of antisites and interstitials such as ${In}_{i}$, $P_{i}$, ${In}_{P}$, and $P_{In}$ can be expressed as follows:

(7) $$E_{f}\left({In}_{i}\right)=E\left({In}_{i},{In}_{N+1}P_{N}\right)-E\left({In}_{N}P_{N}\right)-\mu_{In}$$

(8) $$E_{f}\left(P_{i}\right)=E\left(P_{i},{In}_{N}P_{N+1}\right)-E\left({In}_{N}P_{N}\right)-\mu_{P}$$

(9) $$E_{f}\left({In}_{i}\right)=E\left({In}_{i},{In}_{N+1}P_{N}\right)-E\left({In}_{N}P_{N}\right)-\mu_{In}$$

(10) $$E_{f}\left(P_{In}\right)=E\left(P_{In},{In}_{N-1}P_{N+1}\right)-E\left({In}_{N}P_{N}\right)-\mu_{P}+\mu_{In}$$

In the present work, the chemical potential $\mu_{In}$ and $\mu_{P}$ calculated by Chrobak et al. [21] are used. Meanwhile, the total energies of six supercells containing 512 atoms with single point defect, such as $V_{P}$, $V_{In}$, ${In}_{i}$, $P_{i}$, ${In}_{P}$, and $P_{In}$, are calculated in LAMMPS by using the ABOP + ZBL potential. Then, the total energy $E\left({In}_{N}P_{N}\right)$ of a perfect supercell containing 512 atoms is also calculated with the same method. These values are substituted into the Formulas (5)–(10) to obtain the formation energies of native point defects in InP.

For all DFT calculations in this work, we use the Vienna ab initio Simulation Package (VASP) [37] with projector augmented wave (PAW) potentials and the Perdew–Burke–Ernzerhof (PBE) exchange–correlation functional. The valence electron configurations of 4d^10^5s^2^5p^1^ for In and 3s^2^3p^3^ for P are taken into consideration. The cutoff energy is set as 500 eV, and the Brillouin zone is sampled using a 3 × 3× 3 Monkhorst–Pack k-point mesh for the static calculations.

## 3. Results

### 3.1. Accuracy of Modified Potential

**Figure 1:**
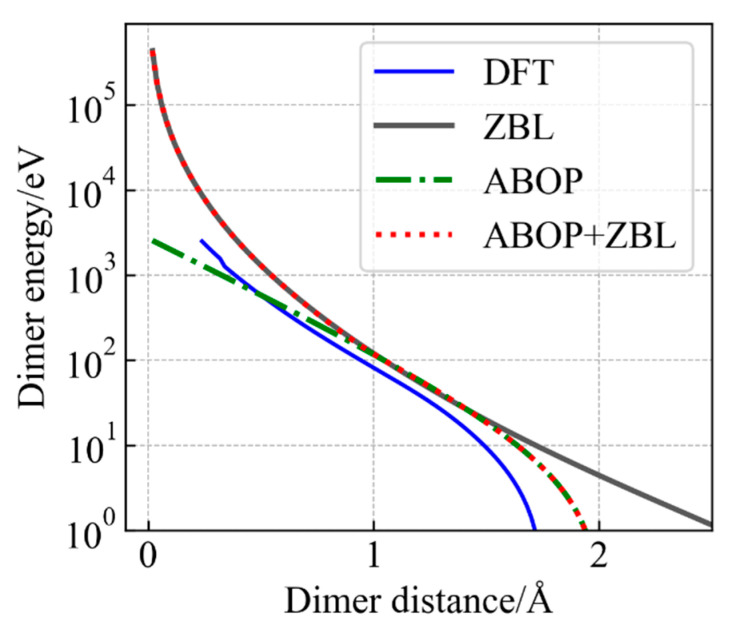
The calculated In–P dimer energy–distance curves based on the DFT method (the blue line), the universal ZBL repulsive potential( the gray line),the ABOP potential (the green dot-dash line) and the ABOP + ZBL potential (the red dotted line).

As shown in Figure 1, the In–P dimer energy–distance curve is calculated by DFT, MD with the ZBL potential, the ABOP potential and the modified potential. It should be noted that due to the Pauli exclusion principle, the repulsive force increases sharply between the small distance pairs in collision cascades. However, the energy predicted by ABOP repulsion interactions is too weak to describe this process. Due to the addition of ZBL, the short-range interactions are well reflected by the ABOP + ZBL potential. The dimer energy of highly close atoms (less than 2 Å) estimated by the ABOP + ZBL potential has a sharp increase, which is close to the value and trend of DFT. Therefore, the modified potential is more suitable for describing primary displacement damage.

As indicated in Table 2, we utilized the ABOP + ZBL potential to calculate the basic physical properties of InP, including the lattice constant, cohesive energy, bulk modulus, and the C_11_, C_12_, and C_44_ elastic constants in the zinc blende structure. The results reveal that there is no disparity in the data values between the ABOP + ZBL potential and the ABOP potential, which are in proximity to the experimental observation [19].

We also investigate the formation energy of defects by using the modified potential. As shown in Table 3, the ABOP + ZBL potential reproduces the formation energy of native point defects in InP, exhibiting excellent agreement with the calculations of DFT and the ABOP potential. These results manifest that the addition of ZBL do not exert any influence on the accuracy of the basic performance calculations of InP.

### 3.2. Quasi-Static Calculations

**Figure 2:**
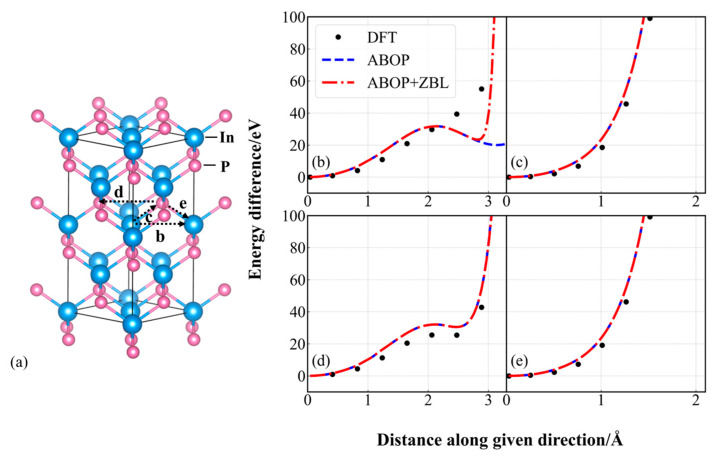
(**a**) Schematic graph of the four representative paths of atomic movement in InP. Total energy difference for quasi-static simulations in InP calculated by DFT (the black points), the ABOP potential (the blue dashed line), and the ABOP + ZBL potential (the red dot-dash line). (**b**) In ⟶ In, (**c**) In ⟶ P, (**d**) P ⟶ In, (**e**) P ⟶ P.

To evaluate the ability of simulating atomic cascade collisions, the quasi-static calculations are simulated by using the ABOP + ZBL potential. In the quasi-static calculations [39,40], one atom is moved towards the nearest atom and the potential energy differences along this direction are calculated by DFT and MD with the ABOP potential and the modified potential. The process provides information on the early stage of a recoil event but in a small system that consists of 216 atoms for comparing the data with DFT. Four representative directions are simulated and the total energy differences along these paths are presented in Figure 2. As illustrated in Figure 2b, within the range of 2~3 Å, the energy difference of the ABOP potential is apparently lower than the DFT value, and the trend of the energy difference calculated by the ABOP + ZBL potential is identical to that of DFT, yet there is a disparity in the value. The reason is that the parameter adjustment of the empirical potential makes sacrifices in some physical performance in order to achieve the global optimal solution. Nevertheless, the calculated value of the ABOP + ZBL potential is proximate to that of DFT in Figure 2c–e, and the maximal difference is merely 41.82 meV/atom, which is within a reasonable scope. The foregoing results reveal that the incorporation of ZBL remedies the shortcomings of the ABOP potential in the short-range interaction force. Moreover, compared with the ABOP potential, the modified potential is more appropriate for simulating the movement of atoms and is more in line with the DFT calculation results.

**Figure 3:**
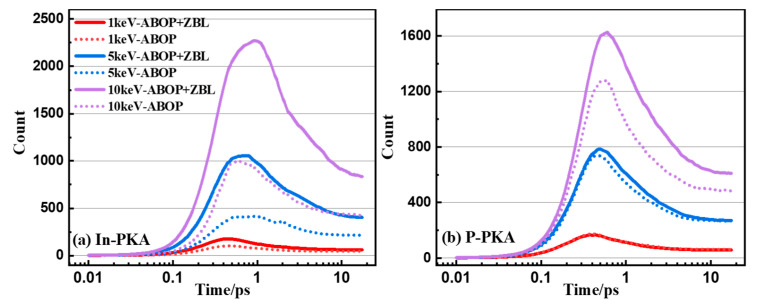
The number of point defects (including FPs and antisites) as the functions of simulation time during the cascade in InP initiated by 1~10 keV (**a**) In-PKA and (**b**) P-PKA. The solid line indicates the results with ZBL and the dashed line indicates the results without ZBL.

It is obvious in Figure 1 that the ABOP potential lacks the description of short-range atomic interaction, so the PKA is able to overcome the ABOP repulsion interactions and is inclined to leave the system rather than produce cascades collision, which results in the underestimate of the displacement damage. However, as shown in Figure 3, the ABOP + ZBL potential can correct the underestimation of the number of point defects calculated by using the ABOP potential, because the addition of ZBL improves the weak repulsion at short distances, which gives a good representation of cascades collision. Furthermore, the ZBL repulsion force is proportional to the atomic mass, which makes the reduction degree constant of the number of point defects induced by In-PKA larger than that of P-PKA. These results suggest that the addition of ZBL has a significant impact on the simulation of cascade collisions and is necessary for obtaining accurate data.

### 3.3. Point Defects

**Figure 4:**
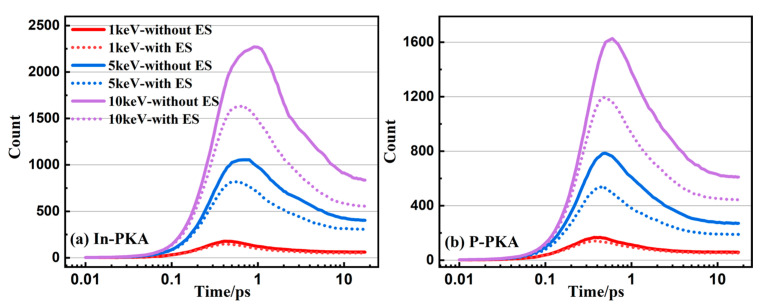
The number of point defects (including FPs and antisites) as the functions of simulation time during the cascade in InP initiated by 1~10 keV (**a**) In-PKA and (**b**) P-PKA. The solid line indicates the results without ES and the dashed line indicates the results with ES.

During the collision cascade of semiconductors, it is well known that there are three basic stages, including a linear collisional stage, a heat-spike or recombination phase, and a remaining primary damage stage. As depicted in Figure 4, the evolution of point defects in InP initiated by 1~10 keV In-PKA and P-PKA follows the same fundamental cascade mechanism. The collisional stage occurs when PKA knocks the lattice atoms out of their normal sites, and the number of point defects increase to the maximum amount within 1 ps to generate the most serious displacement damage. Afterwards, the recombination stage starts, and the displaced atoms move within 10 ps in the systems, which causes the recombination of vacancies and interstitials. The number of point defects becomes stabilized after 10 ps without the migration of important defects which reach the stable damage state in InP. 

As shown in Figure 4, it is obvious that In-PKA causes a greater number of point defects and more severe displacement damage regions than P-PKA, because In-PKA has a larger mass which results in a greater elastic collision cross-section. Additionally, as the PKA energy increases, more time is required to reach the peak number of defects (from 0.4 ps to 0.9 ps for In-PKA, as an example) and the stable damage (from 5.5 ps to 11.5 ps for In-PKA, as an example). This occurs because high-energy PKA generates a larger quantity of defects, leading to a longer annealing time. 

Meanwhile, compared with the number of point defects without ES, ES corrects the overestimation of the number of point defects without ES by reducing the kinetic energy of all atoms produced by the nuclear collisions of PKAs. The reduction rates of the stable defect numbers for 1, 5, and 10 keV In-PKA are 14.5%, 21.1%, and 32.2%, respectively. The reduction rates of the stable defect numbers for 1, 5, and 10 keV P-PKA are 7.9%, 29.2%, and 27.4% respectively. As the energy of PKA increases, the correction effects of ES become more significant. This phenomenon is consistent with the physical rule that a higher kinetic energy leads to greater electron energy loss. Therefore, it is necessary to consider the effect of ES on the displacement damage caused by the high-energy PKA.

**Figure 5:**
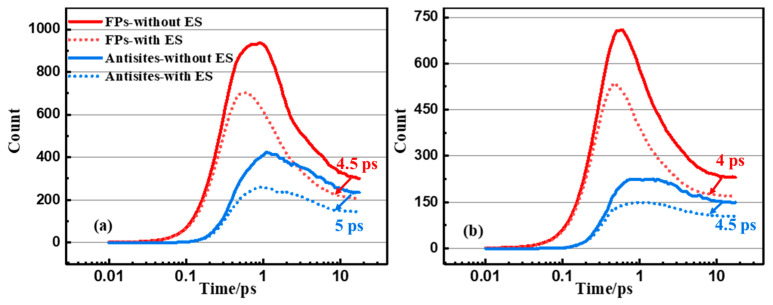
The number of FPs and antisites as the functions of time in InP initiated by (**a**) 10 keV In-PKA and (**b**) 10 keV P-PKA. The solid line indicates the results without ES and the dashed line indicates the results with ES. The arrows represent the change direction of cascade lifetime after considering the ES. The difference of cascades lifetime between without ES and with ES are 4.5 ps, 5 ps for FPs and 4 ps, 4.5 ps for antisites.

In order to further characterize the evolution and nature of these cascades, the number of Frenkel pairs and antisites of InP are plotted in Figure 5 as a function of time for 10 keV In-PKA and P-PKA. The effect of ES leads to a decrease in the number of Frenkel defect pairs and antisites by slowing all atoms down. It is known that a cascade lifetime is the period of time beyond which the variation in final number of defects can be neglected. Compared with the results without ES, the cascade lifetimes of Frenkel pairs and antisites with ES are shortened by 4~5 ps due to the reduction in the number of point defects. For example, in Figure 5a, the cascade lifetime of Frenkel pairs changes from 14.5 ps to 10 ps, while the lifetime of antistites alters from 14 ps to 9 ps. Most of the antisites in InP are generated during the collision stage, and a decrease in the number of antisites occurs during the recombination stage, attributed to short replacement–collision sequences. However, the slow recovery of antisites is discovered for InP, which may be prohibited by the formation of amorphous domains during a cascade. The phenomenon resembles what has been found in SiC [41], GaAs [12], and InAs [18].

**Figure 6:**
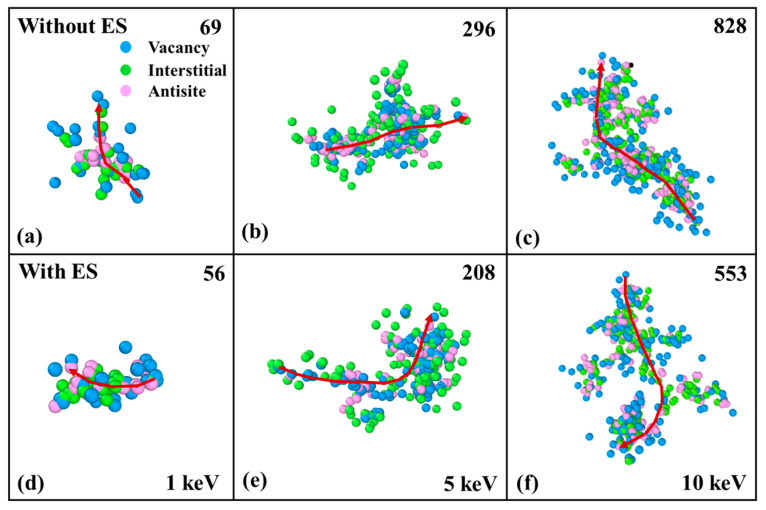
Snapshots of defect spatial distributions at 17.5 ps in InP induced by 1~10 keV In-PKA. The figures (**a**–**c**) present the results without ES and the figures (**d**–**f**) present the results with ES. The blue points are vacancies, the green points are interstitials, and the pink points are antisites. The number in the upper right corner of subfigures represents the quantity of point defects and the red arrow is the trajectory of In-PKAs.

Additionally, the cascade morphology of InP at the final state induced by 1~10 keV In-PKA and P-PKA is also analyzed with and without ES. As depicted in Figure 6a–c, several subcascades are found to form with vacancies, interstitials and antisites in InP along the path of the PKA when PKA’s energy exceeds 1 keV. This feature resembles what has been observed in GaAs [12] and SiC [17]. These subcascades are tightly distributed and prone to the occurrence of incoherence, which causes the degradation of material properties. The spatial distribution of point defects with ES in Figure 6d–f indicates that the electronic energy loss of PKA weakens the generation of the disordered regions, and the majority of surviving defects are single defects and small clusters.

### 3.4. Cluster Defects

**Figure 7:**
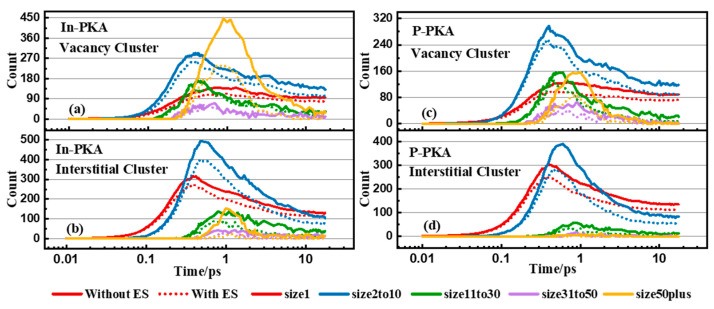
Number of point defects in different cluster size distributions as functions of simulation time in the cascade initiated by 10 keV In/P-PKA: (**a**) vacancy cluster induced by In-PKA, (**b**) interstitial cluster induced by In-PKA, (**c**) vacancy cluster induced by P-PKA, (**d**) interstitial cluster induced by P-PKA. The solid line indicates the results without ES and the dashed line indicates the results with ES.

In addition to point defects, defect complexes (In_P_-V_In_, di-vacancy, di-interstitial, etc.) and large defect clusters significantly affect the macroscopic performance of InP. In this section, we concentrate on the evolution of vacancy and interstitial clusters which are dominant in the cascades because of their low formation energy in InP [10]. For example, the evolution of vacancy clusters and interstitial clusters caused by 10 keV In-PKA and P-PKA are plotted in Figure 7. The classification method of clusters can be seen in Section 2.1. The evolution of vacancy and interstitial cluster shows the same trend. The number of small clusters (containing 1~10 point defects) reaches the peak around 0.4 ps, and then the small clusters decrease and aggregate to form the medium (containing 11~30 point defects) or the large clusters (larger than 31 point defects) within 1 ps. After the recombination of vacancies and interstitials, the number of all-size clusters slows down to stable states and the small clusters are the main component of displacement damage. The influence of ES on the clusters is the same as point defects, and the number of clusters decreases compared to the results without ES.

There is an interesting observation in Figure 7a. Because the high-mass In-PKA makes a large number of lattice atoms move in a short period of time, the large vacancy clusters that contain more than 50 vacancies are found to be the main products around 1 ps. However, the super large vacancy clusters produced by In-PKA quickly quenched within 4 ps for the low migration energy of interstitials in InP [42], which swiftly disperse and recombine with vacancies to reduce their number. Due to the small mass of P-PKA, P-PKA cannot knock out amounts of lattice atoms, and there is no significant increase peak of the vacancy clusters of 50 plus size, as seen in Figure 7c. The migration barriers of interstitials are lower than vacancies in InP [42], which indicates that interstitials can easily move in the InP crystal system rather than accumulate to generate the amorphization, so the small interstitial clusters are dominantly initiated by In-PKA and P-PKA throughout the whole process in Figure 7b,d.

**Figure 8:**
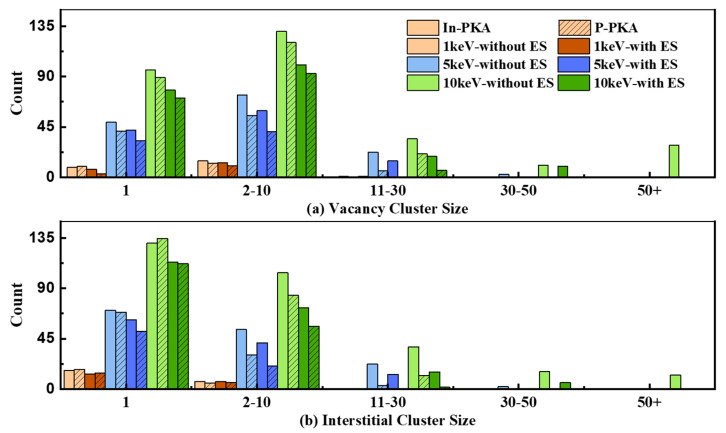
Number of different defect cluster size distributions at the final state of cascades for different PKAs. Solid color blocks mean the defects produced by In-PKA; Twill color blocks mean the defects produced by P-PKA. The different results without/with ES are plotted with different colors.

To further reveal the effects of ES on the primary damage induced by different energies of PKA, the final number of point defects in different cluster sizes generated by 1~10 keV In-PKA and P-PKA are recorded in Figure 8. It is found that the greater the PKA energy, the larger the cluster size that can be generated, but the number of large clusters is low. Due to the mass difference, the number of point defects in different cluster sizes induced by In-PKA is always higher than the results of P-PKA. It is clearly seen that the effects of ES cause the number of medium clusters to extremely decline and even make the large clusters disappear, while the influence of ES only leads to a slight decrease in the number of small clusters. Therefore, the small clusters produced by 1~10 keV In-PKA and P-PKA are predominant products of displacement damage in InP.

## 4. Conclusions

In this work, the effects of electronic stopping on the primary damage of InP induced by 1~10 keV In-PKA and P-PKA were studied using the MD method. To describe the short-range atomic interaction, we modified the ABOP potential of InP by connecting it with ZBL. Moreover, calculations of basic physical parameters and quasi-static demonstrate that the hybrid potential has a good agreement with DFT. By using the modified potential, the effects of electronic stopping on radiation-damage cascades were simulated. Research shows that a smaller number of point defects are produced when the electronic stopping is taken into account, especially for In-PKA. The reduction rates of point defects at stable states are 14.5%, 21.1%, and 32.2% for 1, 5, and 10 keV In-PKA, respectively. Meanwhile, the effects of electronic stopping also lessen the cascades lifetime by about 4~5 ps for FPs and antisites. The corresponding spatial distribution of point defects indicates that the influence of ES results in single point defects and small clusters while the disordered areas are diminished. Lastly, the evolution of clusters and the number of clusters at stable state were analyzed, and the analysis further proved that 1~10 keV In-PKA and P-PKA mainly produce isolated point defects and small vacancy/interstitial clusters (containing 2~10 point defects) in InP. Our study elucidates the evolution mechanism of displacement damage in InP and furnishes a foundation for the research on radiation resistance in InP.

## Figures and Tables

**Table 1:** Cell size setting of MD simulations.

PKA Energy (E_PKA_)/keV	Simulation Box Size (a_0_ = 5.869 Å)
1	$30\;a_{0}$ $\times \;30\;a_{0}$ $\times \;30\;a_{0}$ (21,600 atoms)
5	$50\;a_{0}$ $\times \;50\;a_{0}$ $\times \;50\;a_{0}$ (1,000,000 atoms)
10	$100\;a_{0}$ $\times \;100\;a_{0}$ $\times \;100\;a_{0}$ (8,000,000 atoms)

a_0_ is the lattice constant of InP.

**Table 2:** Basic parameters of InP calculated by different potentials.

Parameter	Experiment [19]	ABOP [21]	ABOP + ZBL
$a_{0}/Å$	5.87	5.83	5.83
$E_{cov}/eV$	−3.34/−3.48	−3.794	−3.794
B/GPa	71.1/72.5	69.1	69.1
C_11_/GPa	101.1/102.2	94.21	94.21
C_12_/GPa	56.1/57.6	56.5	56.5
C_44_/GPa	45.6/46	44.13	44.13

**Table 3:** The formation energy of native point defects in InP.

Defect	DFT [38]	ABOP [21]	ABOP + ZBL	DFT [38]	ABOP [21]	ABOP + ZBL
	In-rich			P-rich		
$\mu_{In},\mu_{P}$	−1.52, −6.94	−1.66, −5.93		−3.96, −4.5	−4.1, −3.49	
$V_{In}$	5.26	5.21	5.24	2.83	2.77	2.8
$V_{P}$	0.98	0.93	0.97	3.42	3.37	3.41
${In}_{i}$	1.62	1.66	1.54	4.06	4.10	3.98
$P_{i}$	4.19	5.81	5.57	1.76	3.37	3.13
$P_{In}$	4.88	4.88	5.13	-	-	-
${In}_{P}$	-	-	-	4.88	4.88	4.89

## Data Availability

The original contributions presented in this study are included in the article material, and further inquiries can be directed to the corresponding author.
